# Introduction to project ECHO (extension for community heathcare outcomes)

**DOI:** 10.1192/j.eurpsy.2021.164

**Published:** 2021-08-13

**Authors:** J. Harrison, M. Leppert

**Affiliations:** 1 Kennedy Krieger Institute, Department Of Psychiatry, Johns Hopkins School of Medicine, Baltimore, United States of America; 2 Kennedy Krieger Institute, Center For Development And Learning, Johns Hopkins School of Medicine, Baltimore, United States of America

**Keywords:** child and adolescent psychiatry, mental health care, mental health disparities

## Abstract

Introduction:

Nationally and internationally, there is a gap between the need and availability of child mental health services. This gap stems from a lack of trained specialists, workforce misdistribution, variations in insurance and financing, and family concerns about the benefits of existing services. Of the possible solutions to these problems, expansion of the child mental health capacity of primary care providers (PCPs) has been proposed as a feasible and scalable approach. The Extension for Community Healthcare Outcomes (ECHO) model was originally conceived and executed by Dr. Sanjeev Arora at the University of New Mexico for Hepatitis C. It serves to de-monopolize medical expertise by extending knowledge from specialists to PCPs

**Objectives:**

After attendance at this session, the learner will be able to: 1. describe the history and expansion of the ECHO model worldwide, 2.name the components and structure of ECHO sessions, 3. discuss ECHO as a force multiplier.

**Methods:**

Dr. Harrison will briefly present the history and expansion of ECHO. She will then describe the program, which consists of a “hub and spokes” model with “tele-clinics” consisting of a “hub” of specialists and “spokes” of clinicians in rural, underserved areas who present cases for discussion, generating treatment recommendations.

**Results:**

The ECHO model has been replicated in a variety of disciplines across the United States and internationally. Its success has been well documented. There are currently 920 active ECHO programs worldwide.

**Conclusions:**

Project ECHO is a viable model to address the workforce shortage of child psychiatrists worldwide.

**Disclosure:**

No significant relationships.

